# Meaningful patient and public involvement in digital health innovation, implementation and evaluation: A systematic review

**DOI:** 10.1111/hex.13506

**Published:** 2022-05-08

**Authors:** Rebecca Baines, Hannah Bradwell, Katie Edwards, Sebastian Stevens, Samantha Prime, John Tredinnick‐Rowe, Miles Sibley, Arunangsu Chatterjee

**Affiliations:** ^1^ Centre for Health Technology University of Plymouth Plymouth UK; ^2^ Patient Experience Library Macfin UK

**Keywords:** codesign, digital health, digital innovation, ehealth, patient and public involvement, systematic review

## Abstract

**Introduction:**

The importance of meaningfully involving patients and the public in digital health innovation is widely acknowledged, but often poorly understood. This review, therefore, sought to explore how patients and the public are involved in digital health innovation and to identify factors that support and inhibit meaningful patient and public involvement (PPI) in digital health innovation, implementation and evaluation.

**Methods:**

Searches were undertaken from 2010 to July 2020 in the electronic databases MEDLINE, EMBASE, PsycINFO, CINAHL, Scopus and ACM Digital Library. Grey literature searches were also undertaken using the Patient Experience Library database and Google Scholar.

**Results:**

Of the 10,540 articles identified, 433 were included. The majority of included articles were published in the United States, United Kingdom, Canada and Australia, with representation from 42 countries highlighting the international relevance of PPI in digital health. 112 topic areas where PPI had reportedly taken place were identified. Areas most often described included cancer (*n* = 50), mental health (*n* = 43), diabetes (*n* = 26) and long‐term conditions (*n* = 19). Interestingly, over 133 terms were used to describe PPI; few were explicitly defined. Patients were often most involved in the final, passive stages of an innovation journey, for example, usability testing, where the ability to proactively influence change was severely limited. Common barriers to achieving meaningful PPI included data privacy and security concerns, not involving patients early enough and lack of trust. Suggested enablers were often designed to counteract such challenges.

**Conclusions:**

PPI is largely viewed as valuable and essential in digital health innovation, but rarely practised. Several barriers exist for both innovators and patients, which currently limits the quality, frequency and duration of PPI in digital health innovation, although improvements have been made in the past decade. Some reported barriers and enablers such as the importance of data privacy and security appear to be unique to PPI in digital innovation. Greater efforts should be made to support innovators and patients to become meaningfully involved in digital health innovations from the outset, given its reported benefits and impacts. Stakeholder consensus on the principles that underpin meaningful PPI in digital health innovation would be helpful in providing evidence‐based guidance on how to achieve this.

**Patient or Public Contribution:**

This review has received extensive patient and public contributions with a representative from the Patient Experience Library involved throughout the review's conception, from design (including suggested revisions to the search strategy) through to article production and dissemination. Other areas of patient and public contributor involvement include contributing to the inductive thematic analysis process, refining the thematic framework and finalizing theme wording, helping to ensure relevance, value and meaning from a patient perspective. Findings from this review have also been presented to a variety of stakeholders including patients, patient advocates and clinicians through a series of focus groups and webinars. Given their extensive involvement, the representative from the Patient Experience Library is rightly included as an author of this review.

## 1 INTRODUCTION

Patient and public involvement (PPI) is frequently cited as a moral obligation,^1^, ^2^, ^3^ with increasing regularity in mandatory policies across a variety of domains including healthcare design and delivery,^4^ research,^5^, ^6^, ^7^ regulation,^8^ education^9^ and perhaps more recently—digital health innovation. While consensus on what to call PPI is not yet available,^10^ its potential benefits are widely acknowledged, including enhanced relevance, quality and authenticity^11^, ^12^, ^13^, ^14^, ^15^; generation of alternative and innovative ideas^13^, ^14^, ^16^, ^17^; stakeholder empowerment^14^, ^18^, ^19^, ^20^; emancipation^21^, ^22^; democratization^23^, ^24^, ^25^; and enhanced sustainability.^11^, ^16^ However, while the COVID‐19 pandemic arguably led to an unprecedented increase in both the innovation and the implementation of digital health technologies,^26^, ^27^, ^28^ this was often at the expense of meaningful involvement,^29^, ^30^ with PPI largely still ‘seen as “nice to have” but not essential’.^30^ ^,^^p.30^ For example, as stated by Richards and Scowcroft,^30^ ‘*The COVID‐19 pandemic saw statutory policy commitments to patient and public involvement and shared decision making in health systems abandoned, the “nothing about us without us” mantra left hanging in the breeze*.^30^,^p.1^

As a result, despite a strong policy rhetoric supported by national agendas such as the UK Long Term Plan and the Digital First Strategy, the extent to which patients and the public are involved in digital health innovations, implementation and evaluation remains largely unknown.^26^ Such findings may have important implications for policy makers, innovators and regulators as evidence suggests that patient involvement in digital health innovation can reduce patient‐related anxieties and reticence to use digital health services^30^ and related technologies. Furthermore, as identified in existing literature,^31^ evidence‐based guidance on how to carry out meaningful PPI in the rapidly evolving field of digital health is lacking, highlighting a further gap in existing knowledge and understanding.

While acknowledging the number of systematic reviews already conducted on PPI in specific fields such as medical regulation,^8^ healthcare services^4^ and research,^6^, ^32^ justification for this review stems from the increasing interest and ‘critical’ need attributed to PPI in digital health innovation^31^, ^33^, ^34^, ^35^; purported lack of attention paid to patient perspectives during digital health innovation design^31^; increasing use of digital health innovations worldwide^36^; and acknowledged importance of working with patients to ensure innovation relevance, value and acceptability.^31^, ^34^, ^37^ Furthermore, while it is widely accepted that digital health technologies should be codesigned,^33^ to the researchers' knowledge, this is the first systematic review of its kind to explore PPI in digital health innovation, implementation and evaluation, highlighting its novel contribution.

The aim of this study was to therefore conduct a systematic review to explore: (i) how, if at all, patients and the public are involved in digital health innovation, implementation and evaluation and (ii) identify factors that affect meaningful PPI in digital health innovation, implementation and evaluation as there may be unique considerations in digital health such as digital skills, patient connectivity and confidence that may be less applicable in other areas of PPI.

The review questions we sought to address were:

1. How, if at all, are patients and the public involved in digital health innovation, implementation and evaluation?
2. What are the barriers and enablers for supporting meaningful PPI in digital health innovation, implementation and evaluation?

In the absence of a single agreed term,^38^ the term ‘patient’ is used to be inclusive of end‐users, clients, service‐users, survivors, citizens, consumers, customers, carers and caregivers. While recognizing the important distinctions between these terms,^39^, ^40^ this decision was made due to ‘patient’ being the most dominant term used in European policy^41^ and previous application in similar research.^10^

## 2 METHODOLOGY

### 2.1 Methods

A systematic review following the Preferred Reporting Items for Systematic Reviews and Meta‐Analysis (PRISMA) guidelines^42^ and Popay's *Guidance on the conduct of narrative synthesis in systematic reviews* ^43^ was conducted.

While recognizing that there is no agreed‐upon definition for digital health, drawing on Fatehi et al.'s^44^ review findings, digital health was defined for the purposes of this review as ‘*the following components of digital health innovation ecosystem: e‐health, m‐health, telehealth and telemedicine, public health surveillance, personalized medicine, health promotion strategies, self‐tracking, wearable devices and sensors, genomics, medical imaging and information systems*’.^44^

### 2.2 Search strategy

#### 2.2.1 Peer‐reviewed literature

The search strategy was informed and approved by an Information Specialist in line with the Peer Review of Electronic Search Strategies guidance.^45^ Search terms (Table 1) were designed to maximize sensitivity and specificity using the SPICE framework.^46^ Reference list searches were also conducted.

**Table 1:** Search terms

S	ehealth or ‘e health’ or e‐health OR ‘digital health*’ OR mHealth OR m‐health OR Telemedicine OR Telehealth OR telecare OR ‘mobile app*’ OR ‘web‐based intervention*’ ‘web based intervention*’ OR ‘internet‐based intervention*’ OR ‘internet based intervention*’ OR ‘wearable*’ OR ‘social robotic*’ OR ‘smart speakers’ OR ‘virtual reality’ OR ‘VR” or ‘augmented reality’ OR ‘AR’ AND design OR evaluation OR implementation OR innovation
P	consumer* OR patient* OR client* OR citizen* OR carer* OR user* OR ‘end user’ OR stakeholder* OR public* OR communit* OR service‐user* OR ‘service user*’
I	involve* OR ‘co‐produc*’ OR coproduc* OR ‘co‐design*’ OR codesign* OR participat* OR engage* OR collaborat* OR ‘experience based design’ OR ‘experience based co‐design’ OR ‘experience based codesign’ OR ‘user‐led’ OR co‐creat* OR cocreat* OR ‘user centered’ OR ‘user centred’
C	N/A
E	N/A

#### 2.2.2 Grey literature

Grey literature was searched via Google Scholar and the Patient Experience Library. Justification for the inclusion of grey literature in this review includes its acknowledged importance in the arsenal of search tools; vital adjunct to traditional database searches^47^; ability to uncover innovative information often in an earlier form following a recognized time lag between research and peer‐reviewed publication; and its ability to potentially minimize bias in a comprehensive search.^47^ Given the rapidly evolving field of digital health innovation, the incorporation of grey literature can be well justified for the purposes of this review.

All review searches were conducted over a 2‐day period (30 June 2020–01 July 2020).

### 2.3 Study selection

Studies were selected through a two‐stage process. First, due to the large number of abstracts returned, five reviewers (RB, HB, SS, KE, JTR) independently examined a 20% share of returned abstracts for study inclusion using predefined inclusion and exclusion criteria outlined below and a collaboratively designed decision flowchart. To ensure consistency, a randomly selected proportion (10%) of each reviewer's abstracts was also blindly assessed and compared by a second reviewer. When an inclusion decision could not be made from the abstract alone, the full article was retrieved. Potentially relevant articles identified through the abstract screening were then read in full and independently assessed for study inclusion by the research team. Any discrepancies that could not be resolved by discussion would have been resolved by being sent to a third reviewer for clarification until consensus was achieved, but this process was not required.

#### 2.3.1 Inclusion criteria

Articles of any study design except for protocols, conference proceedings, letters or theses published in the English language, between 2010 and 2020, that involved patients and/or the public in the innovation, implementation and/or evaluation of digital health technologies were included. Justification for the date parameters used stems from the rapidly evolving nature of digital health technologies and desire to ensure that only the most contemporary information was included.

#### 2.3.2 Exclusion criteria

Protocols, conference proceedings, letters or theses, articles not available in the English language and articles published before 2010 that do not involve patients and/or the public in the innovation, implementation and/or evaluation of digital health technologies were excluded. Due to limited resources, the authors could not ensure a sensitive interpretation of non‐English articles. Non‐English articles were therefore excluded, recognizing that this may have introduced a risk of bias.

### 2.4 Data extraction

Six reviewers (RB, HB, SS, KE, JTR, SP) independently undertook data extraction using a piloted data extraction form to facilitate data extraction consistency. Information extracted included author name, publication date, study location, population and methodology, digital health technology type, stage of involvement, that is, innovation/design, implementation and/or evaluation and reported barriers and enablers.

### 2.5 Data analysis and synthesis

Review findings were analysed using inductive thematic analysis as proposed by Braun and Clarke.^48^ Identified themes were synthesized using a narrative approach, defined as ‘*an approach to the systematic review and synthesis of findings from multiple studies that relies primarily on the use of words and text to summarize and explain findings of the synthesis*’ according to Popay et al.'s^43^ ^,^^p.5^ guidelines.

### 2.6 Registration

The review protocol is published on the PROSPERO website (registration number CRD42020201432).

### 2.7 Patient and public involvement

This review was designed with significant involvement of a patient representative from the Patient Experience Library. Review findings have also been presented in a multistakeholder focus group involving patients, patient advocate organizations, clinicians and digital health innovators as a sense‐checking exercise. The patient representative is rightly included as a coauthor of this review, given their involvement and contributions.

## 3 RESULTS

### 3.1 Descriptive summary

**Figure 1:**
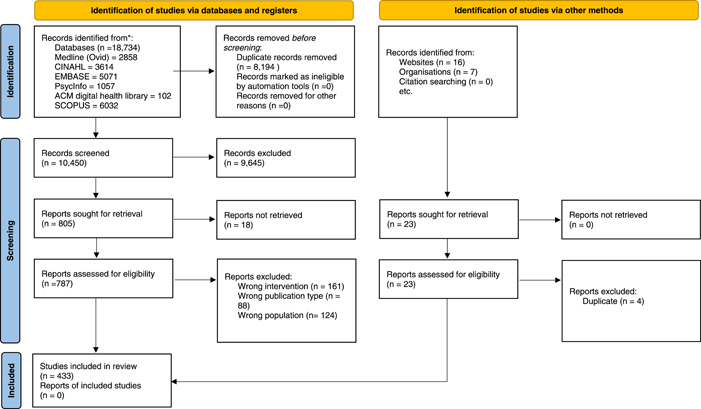
Preferred Reporting Items for Systematic Reviews and Meta‐Analysis diagram

From the 10,540 articles identified, 433 were included (Figure 1). As demonstrated in Material S1, there have been an increasing number of publications over the past decade discussing PPI in digital health innovation, implementation and innovation. For example, in 2010, there were nine identified articles; this figure increased to 109 in 2019. The majority of included articles were published in the United States (*n* = 141), United Kingdom (*n* = 55), Canada (*n* = 33) and Australia (*n* = 26), with representation from 42 countries, highlighting the international prevalence of patient involvement in digital health innovation (Material S2).

A variety of digital health innovations, implementation and/or evaluations were also identified. Those most frequently described included mobile apps (*n* = 172), eHealth interventions (*n* = 52), web‐based interventions (*n* = 24), eHealth resources/sources of information (*n* = 23), robotics (*n* = 17) and online platforms/portals (*n* = 10).

In total, 112 topic areas were identified where PPI had reportedly taken place. Those areas most often described included cancer (*n* = 50), mental health (*n* = 43), diabetes (*n* = 26) and chronic or long‐term conditions (*n* = 19). Other topics of innovation included breastfeeding, aphasia, human immunodeficiency virus and sexually transmitted disease, sleep, hearing loss and impairment.

Interestingly, over 133 terms were used to describe PPI. Terms most commonly used included user‐centred design (with 10 variations identified), participatory design (with 15 variations identified), codesign/cocreation/cocreation (with nine variations identified) or a combination of terminologies (*n* = 47) that were often used interchangeably. Few terms were explicitly defined in included articles, with Norman's user‐centred system design most commonly used.^49^

### 3.2 How, if at all, are patients and the public involved in digital innovation, implementation and/or evaluation?

In response to the first review question, patients were often most involved in the final stages of an innovation journey, for example, usability testing, where the ability to proactively influence change was severely limited. Methods often used to involve patients and the public included think‐aloud interviews, focus groups and surveys.

#### 3.2.1 Perceived importance

Many articles conceded that meaningful PPI is both necessary and fundamental, capable of achieving ‘unexpected consequences’ that are both ‘rewarding and fulfilling’.^50^ For example:

*"The process of involving stakeholders in intervention design and development is fundamental… Interviews with cancer survivors and feedback from health care providers and eHealth experts gave vital direction for intervention design and development. …Even though the user‐centred design process can be labour intensive, time consuming, and as such also costly, it is likely a waste of resources not to invest enough time and effort in the essential design and development phase*."^51^

Similarly, ‘*the stepwise iterative process revealed elements critical to an effective intervention, which otherwise could have been easily missed*’.^52^

### 3.3 Potential benefits

Reported benefits of PPI in digital health innovation, implementation and/or evaluation included improved usability; insight into ‘patients' needs and preferences’^53^; increased ‘*credibility and likelihood of app recommendation and use*’^54^; the development of more *‘equitable adoption and use of interventions by traditionally underserved populations*’^55^; ‘*high levels of satisfaction*’^56^; increased ‘*overall effectiveness of systems*’^57^; ‘*successful implementation*s’^58^; and facilitation of both individual and collective empowerment:

*Target communities are empowered as the strengths and resources within them are identified, harnessed, and showcased. Recognizing the human resources already available within a community, by involving them in the production of their own health education content, serves to validate, educate, and enable that community. Empowered community members can promote health within their peer groups and can advocate for increased access to resources like health and social services on behalf of their communities. An empowered community recognizes and prioritizes health education and the behaviours associated with improved outcomes. They also become valuable partners in the dissemination of their own health education content*.^59^

An ‘*increased sense of awareness, ownership, and identification… by the fact that content better reflects the context, needs and wants of the target community’* was also described as a beneficial outcome of PPI. This increased sense of ownership was also felt to help ‘decrease resistance to health messages (also called counterarguing) as the messages are perceived as coming from sources that have been internally validated, rather than being foreign and external to the community’,^59^ highlighting a further benefit attributed to PPI in digital health innovation.

#### 3.3.1 Reported impacts

Leading on from the benefits outlined above, several authors also reiterated the importance of PPI in digital health innovation and implementation due to its resulting impact. For example, as suggested by Camerin et al.,^57^ ‘*this study empirically confirmed that the adoption of a participatory approach to the design of eHealth interventions and the use of personalized contents enhance the overall effectiveness of systems. Therefore, more time and effort should be invested in involving patients in the preliminary phases of systems' development, maximizing the likelihood to observe the desired effects*’. Similarly, ‘*overall, the findings from this study confirm the importance of including PPI at the early design stage of medical devices*’^60^; ‘*the perspective of person‐centred care helped us apply a broader scope involving the patient as a person in both the process and the final product*’.^61^

### 3.4 What are the barriers and enablers to meaningful PPI in digital health innovation, implementation and evaluation?

#### 3.4.1 Barriers

However, despite a range of reported benefits, the reviewed articles also described a multitude of barriers (Table 2) that prevented or inhibited meaningful PPI in digital health innovation. Barriers most frequently described included time and financial constraints; not being seen as a priority by relevant stakeholders involved; not involving patients early enough in the process; and a disconnect between developers and end users.

**Table 2:** Identified barriers and their implications for meaningful PPI in digital health innovation, implementation and evaluation

Barrier	Implication
Time and resource intensive	Influencing how, when, with who and how often PPI in digital innovation occurs
Not always a priority	Meaning limited time, resources and recognition are directed towards meaningful involvement
Not involved early on	Meaning important design and evaluation decisions including evaluation assessments/criteria are made without patient involvement, jeopardizing product relevance and acceptability
Competing/unaligned timelines	Meaning time for meaningful involvement is not always possible, or as frequent as developers and communities would like
Prolonged design process	Meaning possible delays to market launch, but possible prevention of resource expenditure further down the line
Limited direct contact between developers and patients	Leading to a possible disconnect, meaning decisions made do not always reflect patient requirements, undermining product success and sustainability
Lack of flexibility and open‐mindedness	Limiting the involvement and incorporation of patient insights
Balancing competing demands and priorities	Leading to potential delays or disengagement if patients do not feel listened to and heard
Use of ineffective methods	Meaning methods used to support innovation design and/or evaluation are ineffective or inappropriate for meaningful PPI, with some suggestions that existing methods are often ‘developer‐focused’ as opposed to ‘patient‐centred’
Extensive exclusion criteria	Often means that design decisions and evaluations are based on an intentionally, often healthy, selected proportion of the total target population. People affected by mental health, individuals with limited literacy levels, low levels of education or smartphone ownership appear to be disproportionately affected
Limited interoperability or IT governance systems	This can prevent involvement opportunities and lead to a potential biasing of innovation and evaluation responses/decisions
Recruiting a ‘sizable’ and representative sample	This can be hard, particularly when working with individuals from ‘seldom‐heard’ communities, but is often considered essential in ensuring product relevance, cultural sensitivity and acceptance. Patient confidentiality can also make it difficult for developers to recruit from active patient lists
Maintaining interest and preventing dropout rates	This can be challenging, particularly when working with ‘oversaturated’ or fatigued communities, or people likely to experience deteriorating health such as when working with people with dementia. However, maintaining levels of interest is considered essential for maintaining continuity and reducing possible bias
Discussing sensitive/personal topics in group settings and unequal opportunities to take part in group sessions	This can prevent equitable and authentic involvement, with some patients withholding information that may be integral to innovation design, implementation and/or evaluation. The presence of carers and/or parents was also felt to prevent some people from being honest in their responses
Data privacy, security and trust	Failure to establish trust and provided assurances of data privacy and security can affect people's willingness to be involved and inhibit product acceptance
Bias	A number of biases can be introduced into PPI initiatives including the setting in which PPI activities are undertaken, recruitment methods, for example, primarily all online, preventing involvement from those considered to be digitally excluded, volunteer bias (people who opt in or take part in research/innovation may have greater interests/motivations and may therefore present the perspective of early adopters only), sharing existing digital innovations/ideas before data collection inducing possible response bias, previous involvement in digital innovation creation when evaluating the product leading to possible social desirability bias, exclusion from data analysis and shortened evaluation times that may present an overinflated experience
Practical difficulties including reimbursement, identifying times when everyone is free, not recording involvement sessions, not articulating why it may be beneficial to get involved, not considering the cost of downloading required content, for example, data charges, and sharing incorrect contact information	This can significantly hinder PPI activities and experience and collection of meaningful insights

Abbreviation: PPI, patient and public involvement.

##### 3.4.1.1 Time and financial resources

PPI in digital health innovation was often described as time and resource intensive, affecting PPI occurrence and duration. For example, ‘*time and resource constraints will inevitably influence how the intervention planning process can be carried out, and when these are limited, it will only be possible to engage in rapid, “light touch” evidence collation and theoretical modelling including the target group*’.^62^ As a result, patient involvement did not always happen as much as was desired—‘*due to time and financial constraints, it was not always possible to involve users as much as we would have hoped*’.^63^ Several articles also repeatedly acknowledged that the ‘*timeline of developers, academic and funders often do not match the communities*’,^64^ causing further complications and frustrations. Similarly, project ‘*timelines did not always align with the speed of coproduction*’.^65^

##### 3.4.1.2 Disconnect between design processes and market launches

Other barriers attributed to PPI in digital innovation included a possible disconnect between the time taken to undertake PPI and product market launch. For example, ‘*the whole intervention development process took place over a 4‐year period, which is quite time consuming and could increase the risk of a misfit with current market developments or that technology has moved on by the time of implementation*’.^66^ However, several articles suggested that meaningful PPI in the design stages could minimize resource expenditure further down the line, warranting its adoption. For example, ‘*although the aim of this intervention development was to apply a collaborative approach, this resulted in an extended development period… On the other hand, engaging clinicians and patients in the design phase can minimize problems and delays during implementation*’.^67^

##### 3.4.1.3 Balancing competing demands and priorities

Similar to prolonging the design process, some articles also recognized the difficulty of reconciling competing demands or priorities, with potential ramifications for implementation success and resource expenditure if they remained unaddressed. For example, ‘*a further issue is the continuous reconciliation between desires, preferences and needs of different kind of users and technology that imposes limits on the cost efficiency of the product. Involving users requires to agree on every decision on system development by establishing balances among user needs and desires, technology possibilities and costs. Meanwhile avoiding users in such technological decisions results in increasing the refusal rate of the final product, involving them requires an additional effort…*’.^68^ Meaningful PPI was also described as a ‘challenging’, or difficult thing to do, often increasing the perceived complexity of the design process, but not necessarily the complexity of the resulting product/outcome.

##### 3.4.1.4 Lack of flexibility and open‐mindedness

Some articles reported lack of flexibility or open‐mindedness from designers, funders, researchers and/or their institutions, including ethical procedures and requirements. As suggested by Lustria et al.,^69^ ‘*the design team has to be careful not to be constrained by their initial ideas for the design and keep in mind the needs of each user and the realities of their work settings while also allowing for the variety of users, their information needs, and their assumptions about how a such a system should—and does—work*’. Similarly, it could be argued that the purpose of involvement is to respond to patient input as it emerges throughout the innovation journey. This is often at odds with gaining ethical approval, with committees often needing to know what will exactly be done, how, by who and when. Difficulties related to ethical approval processes are often universal, but appear particularly problematic in relation to codesign and implementation research.^70^

##### 3.4.1.5 Disconnect between developers and patients

Other areas of contention included a disconnect between developers/researchers and patients, meaning that decisions made did not always reflect patient requirements, often defaulting to developer or researcher assumptions and preferences. PPI also often required developers to work in a way they were not ‘used to dealing with’. For example, ‘*the other stage of the design process was related to the dialogue between the researchers who collect empirical data and the technical partners who develop the system who are not in contact with the end‐users. In particular, we can report that personas and scenarios were not enough to ensure an efficient dialogue between stakeholders. It was difficult to convince technical partners that they should focus on personas when developing the application. Also scenarios looked too narrative for them compared to functional specifications that they were used to dealing with*’.^71^

##### 3.4.1.6 Power

Linked to concerns of inflexibility and the requirement of working in new ways was the concept of power, specifically, the retention of power by researchers and/or digital innovators both in terms of PPI design, frequency and duration, but also who has the final say. One article by Buus et al.^72^ described this issue at length:

Researchers controlled most of the concrete user‐involving processes… it remained debatable to what extent the software developers and researchers were committed to collaborate and genuinely share control. For example, the software developers were adamant in maintaining X despite user dissent. In addition, although study participants were consulted over extended periods of time… the researchers ultimately controlled the data‐collection sessions and the information that was recorded, prioritized, and fed back to the software developers and programmers.^72^

Some authors also described an inherent power imbalance within the current system and culture that still largely remains paternalistic. Hesitancy to adopt participatory design may therefore stem from an existing culture or current ways of working that do not appropriately acknowledge or respond to the importance of PPI and its historical context:

Reducing the participant to an informant is a potential risk in a paternalistic healthcare system, which is a remnant from the past, where there traditionally is little room for the patient's wishes and where involvement is only superficial. This highlights the importance of understanding the conditions and consequences of including users in designing technology, as well as the selection of the proper methods for genuine participation; otherwise the participation will not be genuine… The challenges associated with participatory design when applied in different healthcare systems and settings include the existing power distributions, language and culture among those who work there, where involvement is nothing near the genuine participation that characterizes participatory design.^73^

##### 3.4.1.7 Data privacy, security and trust

Unsurprisingly, concerns around data privacy and security also appeared to affect people's trust and willingness to be involved in digital health innovation, implementation and/or evaluation. Patients often required assurances that their personal data would not be accessible to any unauthorized persons or organizations. For example, ‘*we have encountered a limitation in communicating with patients about the protections in place for patient privacy and confidentiality. Care managers are the first‐line providers who field patient questions; however, they do not have technical expertize in the system and therefore have needed additional support in responding to patients' technical questions related to data transmission and security*’.^74^

Often linked to conversations around data privacy and security was trust, something considered to be particularly difficult to establish when communicating online or discussing digital health innovations that involved data sharing online. Trust, particularly in relation to data sharing, was repeatedly described as imperative in encouraging meaningful patient involvement and engagement and may be an important area for future exploration. Ensuring that everyone involved in communicating digital innovations to patients and the public is well supported and informed about data privacy and security also appears to be essential.

##### 3.4.1.8 Use of predetermined evaluation tools with limited or no patient involvement

Other areas of difficulty described included the use of predetermined evaluation tools with limited or no patient involvement. This often meant that aspects of digital innovations or evaluations considered to be of most importance from a patient perspective were not included, often favouring more technical or functional specifications, as opposed to broader definitions of ‘success’. For example, ‘*the survey questionnaire was drafted based on a survey from existing mHealth‐related literature, and the final version was completed after review and discussion by a group of experts, including two doctors from a cardiology department, one medical informatics professor, one nurse, two researchers, and three developers*’.^75^

##### 3.4.1.9 Discussing sensitive topics in a group setting and concerns of confidentiality

Linked to concerns of privacy and trust were also concerns of the safety and privacy of information shared during focus groups discussions, particularly when discussing sensitive topics. For example, as discussed by Chhoun et al.,^76^ ‘*there are a few limitations that are important to note. First, in order to capture a wide variety of opinions and to generate rich dialogues, we utilized focus group discussions; however, we recognized that for issues such as gender‐based violence and substance use, these may not promote enough confidentiality and privacy to allow for full disclosure of experiences. There may be issues that women are reluctant to discuss in a group, but may feel comfortable opening up about in an interview setting*’. While this may reflect a methodological limitation in choosing an inappropriate or insufficient method, it is an important point to consider for both developers and researchers when working with patients and the public.

##### 3.4.1.10 Lack of early involvement

Finally, failing to involve patients early enough in the design stage was a frequently reported barrier that often underpinned many other difficulties encountered. For example, ‘*children could have been involved at an earlier stage of the project, which would have allowed their participation in the planning process*’.^77^ In many cases, patients were not involved at all in the early stages of digital design. Despite this, early involvement was felt to be critical in preventing unnecessary resource expenditure and, importantly, reducing innovation refusal rates: ‘*involving users requires to agree on every decision on system development by establishing balances among user needs and desires, technology possibilities and costs. Meanwhile avoiding users in such technological decisions results in increasing the refusal rate of the final product, involving them requires an additional effort to close them to technology reality*’.^68^

#### 3.4.2 Enablers

Although less frequently discussed, some articles also described several facilitators (Table 3) for supporting meaningful PPI in digital health innovation, with each enabler often designed to counteract the barriers identified above.

**Table 3:** Suggested enablers to support meaningful PPI in digital health innovation, implementation and evaluation

Suggested enablers	Supporting quotes
Commit to sharing power, working in equal partnerships that treat insights equally, irrespective of their source	Specifically ‘better balancing the power relations that exist’^71^; ‘a democratic partnership with appropriate distribution of power’,^76^ or ‘in bidirectional equitable partnerships’.^77^ Similarly, ‘the methods for the design expressly included patients and staff with all voices treated equally and regarded as key contributors to design’.^49^
Involve patients early on	‘Findings from this study confirm the importance of including PPI at the early design stage of medical devices’.^59^
Work in an interactive, open‐minded and adaptive manner	‘The whole process required flexibility, an open mind, and a willingness to revise material iteratively’.^78^ Similarly, ‘it was necessary to take a highly iterative approach’.^75^
Work to establish trust	‘It was critical to ensure timely and consistent follow‐up in response to any technical or personal issues that are reported by the participants. This is an important part of building participants' trust in the intervention and the staff. Trust of the programme and trust of outreach workers was a priority issue, which needed to be addressed during all aspects of the programme roll‐out. It was helpful to brand the Mobile Link programme and have Cambodian government buy‐in so that women know that the programme they are signing up for is medically accurate and trustworthy’.^74^
Be sensitive to people's spiritual, religious and cultural beliefs/values	Considering peoples ‘spiritual, religious and families values when designing digital health innovations’^63^ is imperative, particularly when working with indigenous and Hispanic communities.
Create engaging activities	‘It is important to ensure that the methods and user activities fully engage the participants’^71^
Communicate clearly, regularly and inclusively in an age‐appropriate and developmentally appropriate way, including the perceived benefits of taking part	‘A developmental or age‐appropriate approach is needed regarding the content and design of a programme, and accounting for the range of interests and tastes’.^76^ Suggested use of *‘*glossaries, use of visual aids/picture topic clues, and videos to facilitate understanding of information’.^79^ ‘Full commitment requires motivating the participants and convincing them about the usefulness of the project, which proved to be more complicated than we thought. The primary motivating factor was their feeling of participating in the creation of services for the future’.^69^
Offer people involved a choice of communication methods	For example, ‘WhatsApp was a significant production asset, useful in soliciting feedback from community members who did not regularly use email and did not feel comfortable editing scripts using Google Drive’.^58^
Clarify people's roles, decision‐making processes and manage expectations	Creating a ‘memorandum of understanding’^77^ and generating ‘ground rules’^80^ or ‘rules of conduct’^81^ were identified as helpful ways to clarify roles and manage expectations. Other suggestions included ‘knowing each others role in the relationship’^82^ and clarifying to partners involved ‘that their individual wishes will not always be met’ (83).
Provide clear instructions, tech support and relevant device access	‘The elderly can be insecure because they are afraid of doing something wrong, so giving clear directions and affirmation is important. They also often need repeated explanations and daily training or courses in learning how to use a tablet for instance’.^69^
	‘Members of the research team set up patients' phones and supported them throughout the study. Phones with the app pre‐loaded were available on loan for people without an Android phone’ (84).
	‘Provide a hotline in case of technical difficulties’ (85).
Allow time for people to become familiar with the tech	‘Given access for a minimum of 2 months to allow sufficient time to work through the programme’.^76^
Work with local organizations to facilitate recruitment	‘One of the core principles of patient participation relates to ensuring that engagement is made as easy, feasible and as flexible as possible… With these goals in mind, it was deemed that participant recruitment through a familiar agency… would be optimal’.^59^ Other ways of facilitating recruitment included recruiting through existing patient lists or ‘established patient groups’ (86).
Acknowledge people's time	Articles described a range of ways to acknowledge peoples time including prize draws, certificates of attendance/achievement, education credit, gift cards (ranging from $5–$100 depending on time spent and level of activity), shopping vouchers and grocery cards.
Encourage developers and patients to work together in the same room	‘We also recommend organizing meetings between developers and users, like test sessions during which the developers would be present to see for themselves the ways in which end‐users actually use the technology. Another option would be to use video in order to show developers the reactions of the end‐users when interacting with the devices and application’.^69^
Create a safe space for people to share their thoughts and ideas	‘We put the primary focus on ensuring all stakeholders felt a part of the process and opened up about their experiences without feeling judged. During all phases, we highlighted the importance of anonymity for this purpose and thus did not collect the demographic information of the participants’ (87); ‘Each session took place at a convenient venue (e.g., community clinic) on weeknights, ranged from 90 to 120 min, and was audio recorded. Before each session, participants shared a meal and informally discussed community health and events. All meetings began with an opening prayer by church leadership to set an atmosphere of creativeness, inspiration, and togetherness among the attendees’ (88).
Hold activities in suitable locations	‘Interviews were held either at Cardiff University or a location convenient for the participant (e.g., home and school). During the interviews, young people stated they were able to discuss the programme openly and appreciated that they could choose the location, and whether they were seen with their parents or carers’.^76^
Provide people with a choice of how and who they would like to be involved	‘Young people were asked whether they would like to be interviewed alone or with a parent or carer. The parent or carer was also asked whether they would like to be interviewed separately’.^76^

Other suggested facilitators included communicating who has the final design say with two divergent approaches available: (i) those that are patient led and (ii) those that are researcher/designer led; allow sufficient time and flexibility to account for unexpected delays and setbacks; create a feedback loop to help facilitate ongoing engagement and generate confidence in the innovation process; provide a named point of contact within the team responsible for maintaining contact; and reimburse expenses such as data charges, travel, childcare and parking in a timely manner.

Other areas of consideration included avoiding overburdening patients with activities and requirements; working with trained facilitators, either recruiting a professionally trained facilitator who ideally spoke the local/native language and understood local cultural and spiritual beliefs, or supporting a local community member through relevant training to facilitate design/evaluation discussions; and having a clear plan to resolve competing demands, particularly if sufficient resources were not available. Suggested plans included capturing the feedback shared and sharing this ‘with developers for later in the platform's roadmap and developments’.^74^ This way, important insights and suggestions were still recorded.

## 4 DISCUSSION

This review addresses an identified gap in the existing literature by exploring how, if at all, patients and the public are involved in digital health innovation and the key barriers and enablers that affect meaningful involvement in digital health innovation, implementation and evaluation.^26^, ^31^ While acknowledging the number of systematic reviews already conducted on PPI in different domains,^4^, ^6^, ^8^, ^32^ this review is the first of its kind (to the researchers' knowledge) to advance current understanding of PPI in digital health innovation in particular.

Key findings from this review include the acknowledgement that there have been an increasing number of patient involvement‐focused publications on a wide range of digital health innovation types and topics including mental health, dementia and cancer over the past decade. However, despite the range of benefits reported and strong policy rhetoric, patients are rarely involved from the outset of digital health innovations, with involvement opportunities often confined to the later stages of usability testing, where the ability to proactively influence change is severely limited. Few articles described the early involvement of patients and the public in the initial design, or idea generation stages of digital innovations. Several barriers and enablers affect the quality, frequency and duration of PPI in digital health innovation, including concerns over data privacy and security, time and financial constraints and an unequal distribution of power, further hindered by traditional, often hierarchical ways of working, with patient insights and suggestions often seen as inferior during the innovation and implementation process.

Perhaps unsurprisingly, many of the review findings align with the existing literature including the importance of partnership working, communicating clearly, regularly and inclusively and sufficiently resourcing PPI endeavours.^10^ Other areas of similarity include the multiplicity of terms used to describe patient involvement, with over 133 terms identified in this review.^10^ Few terms were explicitly defined in the included articles. However, given its particular focus on digital health innovations, unique findings of this review include the importance attributed to providing assurances of data privacy and security, device access, technology support and instructions and allowing sufficient time for people to become familiar with the digital health innovations under review. Identification of these reportedly unique aspects of supporting meaningful PPI in digital innovation further accentuates the novel contribution of this review.

### 4.1 Strengths and limitations

The strengths of this review include its development with an Information Specialist, application of an internationally recognized systematic review process^78^ and incorporation of grey literature, considered to be a vital adjunct to traditional database searches,^47^ given its ability to uncover innovative information in an earlier form and codesign with patient representation from the outset. The integration of a previously disparate literature that remains a growing area of international interest (i.e., PPI in digital health innovation, implementation and innovation)^36^ into one singular source of information is also considered to be a particular strength of this review, given the increasing interest and critical need attributed to PPI in digital innovation.^31^, ^33^, ^34^, ^35^ Furthermore, the extensive results uncovered may be indicative of the extensive search strategy undertaken and adoption of inductive thematic analysis, avoiding the use of predefined and potentially restrictive frameworks. However, the limitations of this review must also be acknowledged. While a rigorous search strategy was used, this study included English‐language articles only. The possibility of publication bias is therefore recognized. Similarly, the findings from this review are reliant on the quality of information presented in reviewed articles. Descriptions of PPI have previously been described as highly variable with regard to quality.^79^, ^80^

### 4.2 Implications

Implications of this review include those related to policy, practice, regulation and research. First, more efforts should be made to support innovators and patients to become meaningfully involved in digital health innovations from the outset, given their reported benefits and impacts including reducing patient‐related anxieties^30^ and improving innovation relevance, value and acceptability.^31^, ^34^, ^37^ Second, critical consideration of existing methods and approaches used to support patient involvement in digital health innovation is required. Many articles reviewed typically relied on passive methods including surveys and questionnaires, where the ability to influence change was severely limited. More creative methods that enable patients and innovators to voice their suggestions and ideas in their own words, as opposed to those that have already been defined for them should be used. This second implication requires a true commitment to sharing power from digital health innovators, researchers and regulators, recognizing that the participatory or codesign process may help to address existing power imbalances, particularly when tailored to individual needs and cultural sensitivities of individual community groups. Similar to patient involvement and coproduction in research, efforts are often unsuccessful due to ‘structural inequalities… that impede coproduction’.^81^ ^,^^p.2^ As such, there is a need to question the extent to which meaningful PPI can truly operate on an equal footing in digital health innovation if it is to achieve its egalitarian and utilitarian potential.^82^

Other implications of this review include the importance of identifying ways to establish trust and transparency amongst patients and the public in digital health innovations, particularly with regard to data privacy and sharing. Establishing trust around these areas is often integral to innovation success and adoption. Finally, building on the findings of this review, gaining consensus on the principles that underpin meaningful involvement in digital health innovation and implementation and evaluation from a variety of stakeholders across the digital health innovation ecosystem including patients, innovators and clinicians would be invaluable in helping to further advance our current knowledge and understanding of this important topic.

## 5 CONCLUSION

PPI is largely viewed as valuable and essential, but rarely practised in the design, implementation and evaluation of digital health innovations. A number of barriers exist for both innovators and patients that currently limit the frequency, quality and duration of PPI in digital health innovation, although clear improvements have been made in the past decade. Some reported barriers and enablers such as the importance of data privacy and security appear to be unique to PPI in digital health innovation, implementation and evaluation. Multi‐disciplinary consensus on the principles and practicalities that underpin meaningful PPI in digital health innovation would be invaluable, as would suggested solutions on how to overcome some of the most common barriers identified.

## AUTHOR CONTRIBUTIONS

Rebecca Baines conceived and planned the review. Rebecca Baines and Sebastian Stevens ran the database searches once approved from the information specialist. Miles Sibley ran the searches in the Patient Experience Library. Hannah Bradwell, Katie Edwards, Sebastian Stevens, John Tredinnick‐Rowe and Sebastian Stevens were all involved in abstract reviewing and data extraction. All authors discussed the results and contributed to the final manuscript.

## CONFLICTS OF INTEREST

The authors declare no conflicts of interest.

## Supporting information

Supporting information.

Supporting information.

## Data Availability

The data that support the findings of this study are available from the corresponding author upon reasonable request.
