# Supplementary material for: Meaningful patient and public involvement in digital health innovation, implementation and evaluation: A systematic review
Source: Health Expect. 2022 May 8;25(4):1232–45. doi: 10.1111/hex.13506 (PMC9327849; doi:10.1111/hex.13506)
Supplement: Supplementary file 1 — Supporting information. [file HEX-25--s001.docx]

| Year of publication | Number of included articles |
| --- | --- |
| 2010 | 9 |
| 2011 | 9 |
| 2012 | 9 |
| 2013 | 10 |
| 2014 | 20 |
| 2015 | 22 |
| 2016 | 48 |
| 2017 | 54 |
| 2018 | 83 |
| 2019 | 109 |
| 2020 | 60 |
