# Supplementary material for: Meaningful patient and public involvement in digital health innovation, implementation and evaluation: A systematic review
Source: Health Expect. 2022 May 8;25(4):1232–45. doi: 10.1111/hex.13506 (PMC9327849; doi:10.1111/hex.13506)
Supplement: Supplementary file 2 — Supporting information. [file HEX-25--s002.docx]

| Country | Number of included articles |
| --- | --- |
| US | 141 |
| UK | 55 |
| Canada | 33 |
| Australia | 26 |
| Multi-site | 21 |
| Netherlands | 18 |
| Sweden | 15 |
| Denmark | 13 |
| Norway | 12 |
| Germany | 9 |
| Spain | 8 |
| New Zealand | 7 |
| Ireland | 6 |
| Switzerland | 6 |
| China | 5 |
| Taiwan | 4 |
| France | 3 |
| Columbia | 3 |
| South Africa | 2 |
| Singapore | 2 |
| Greece | 2 |
| Indonesia | 2 |
| Malaysia | 2 |
| Rwanda | 2 |
| Iran | 2 |
| Chile | 2 |
| Italy | 2 |
| Cambodia | 2 |
| Africa | 1 |
| Republic of Korea | 1 |
| South Korea | 1 |
| Cyprus | 1 |
| Turkey | 1 |
| Portugal | 1 |
| Japan | 1 |
| Argentina | 1 |
| Burkino Faso | 1 |
| Malawi | 1 |
| Tanzania | 1 |
| Austria | 1 |
| Brazil | 1 |
| Mexico | 1 |
| Missing/unclear | 14 |
